# Smoking characteristics and years since quitting smoking of US adults diagnosed with lung and bladder cancer: A national health and nutrition examination survey analysis

**DOI:** 10.1590/S1677-5538.IBJU.2023.0625

**Published:** 2024-03-18

**Authors:** Edoardo Beatrici, Muhieddine Labban, Dejan K. Filipas, Benjamin V. Stone, Leonardo O. Reis, Filippo Dagnino, Giovanni Lughezzani, Nicolò M. Buffi, Stuart R. Lipsitz, Timothy N. Clinton, Richard S. Matulewicz, Quoc-Dien Trinh, Alexander P. Cole

**Affiliations:** 1 Brigham and Women's Hospital Harvard Medical School Division of Urological Surgery and Center for Surgery and Public Health Boston MA United States Division of Urological Surgery and Center for Surgery and Public Health, Brigham and Women's Hospital, Harvard Medical School, Boston, MA, United States;; 2 Humanitas Research Hospital Department of Urology Milan Italy Department of Urology, Humanitas Research Hospital – IRCCS, Milan, Italy;; 3 University Medical Center Hamburg-Eppendorf Department of Urology Hamburg Germany Department of Urology, University Medical Center Hamburg-Eppendorf, Hamburg, Germany;; 4 Universidade Estadual de Campinas Faculdade de Ciências Médicas Campinas SP Brasil UroScience, Faculdade de Ciências Médicas, Universidade Estadual de Campinas, UNICAMP, Campinas, SP, Brasil;; 5 Pontifícia Universidade Católica de Campinas Faculdade de Ciências da Vida Divisão de Imuno-Oncologia Campinas SP Brasil Divisão de Imuno-Oncologia, Faculdade de Ciências da Vida, Pontifícia Universidade Católica de Campinas, PUC-Campinas, Campinas, SP, Brasil;; 6 Brigham and Women's Hospital Harvard Medical School Department of Medicine Boston MA United States Department of Medicine, Brigham and Women's Hospital, Harvard Medical School, Boston, MA, United States;; 7 Memorial Sloan Kettering Cancer Center Department of Surgery and Department of Urology New York NY United States Department of Surgery and Department of Urology, Memorial Sloan Kettering Cancer Center, New York, NY, United States

**Keywords:** Urinary Bladder Neoplasms, Smoking Cessation, Risk

## Abstract

**Purpose:**

Smoking is a recognized risk factor for bladder BC and lung cancer LC. We investigated the enduring risk of BC after smoking cessation using U.S. national survey data. Our analysis focused on comparing characteristics of LC and BC patients, emphasizing smoking status and the latency period from smoking cessation to cancer diagnosis in former smokers.

**Materials and Methods:**

We analyzed data from the National Health and Examination Survey (2003-2016), identifying adults with LC or BC history. Smoking status (never, active, former) and the interval between quitting smoking and cancer diagnosis for former smokers were assessed. We reported descriptive statistics using frequencies and percentages for categorical variables and median with interquartile ranges (IQR) for continuous variables.

**Results:**

Among LC patients, 8.9% never smoked, 18.9% active smokers, and 72.2% former smokers. Former smokers had a median interval of 8 years (IQR 2-12) between quitting and LC diagnosis, with 88.3% quitting within 0-19 years before diagnosis. For BC patients, 26.8% never smoked, 22.4% were active smokers, and 50.8% former smokers. Former smokers had a median interval of 21 years (IQR 14-33) between quitting and BC diagnosis, with 49.3% quitting within 0-19 years before diagnosis.

**Conclusions:**

BC patients exhibit a prolonged latency period between smoking cessation and cancer diagnosis compared to LC patients. Despite smoking status evaluation in microhematuria, current risk stratification models for urothelial cancer do not incorporate it. Our findings emphasize the significance of long-term post-smoking cessation surveillance and advocate for integrating smoking history into future risk stratification guidelines.

## INTRODUCTION

Bladder cancer (BC) is the most common smoking-related genitourinary cancer (1, 2). The duration and intensity of tobacco exposure has a strong relationship with BC incidence and potentially disease stage at presentation. Active smokers have been identified as the individuals at greatest risk of developing BC, with a two to four-fold higher risk than never smokers (2–5). Since 2005, in the US and worldwide, the prevalence of former smokers has grown to exceed active smokers, potentially due to effective anti-smoking campaigns (6, 7).

It is not currently known how the risk of developing BC changes over time among those that quit smoking. By way of comparison, it has been shown that the risk of developing lung cancer (LC) is greatly reduced 15 years after quitting smoking. Accordingly, the US Preventive Services Task Force (USPSTF), former smokers aged 50-80 years no longer need thoracic CT scans after this point (8). There is comparatively less data about how the risk of BC changes after quitting smoking. Still, there is evidence that the risk of BC might persist up to several decades (9–12). Thus, we hypothesized that the risk of BC might persist over several decades even after quitting smoking. In this setting, we sought to assess differences in the smoking status and the interval from quitting smoking to cancer diagnosis among a national sample of men and women with a personal history of LC and BC.

## MATERIALS AND METHODS

### Data source

The National Health and Nutrition Examination Survey (NHANES) is a major National Center for Health Statistics program designed to assess adults’ and children's health and nutritional status in the US by combining interviews and physical examinations. This nationally representative cross-sectional survey is conducted in two-year cycles, and data are available to the public on the National Center for Health Statistics website (13). The survey uses a complex, multi-stage, stratified sampling frame design to ensure that the sample is representative of the US civilian, non-institutionalized population of all ages. The sample is updated every two years to maintain representativeness. Data are collected during an in-home interview and in a mobile examination center. In the in-home interview, the family and sample person questionnaires are administered by trained interviewers in English or Spanish using the Computer-Assisted Personal Interview (CAPI) system. The overall response rate is approximately 60-70%, varying across demographic groups and survey cycles. All the data are then reviewed and edited to guarantee completeness and consistency of answers, and the resulting data are weighted to address non-response bias (14, 15). We obtained institutional review board approval (IRB Number: 2015P000341) for the use of de-identified administrative data.

For this study, we retrospectively queried NHANES to collect data from successive survey cycles conducted between 2003 and 2016. Specifically, we focused on extracting data from the “Questionnaire data”, “Medical conditions” and “Smoking-Cigarette Use” sections, derived from the in-home interview of the survey. It is pertinent to note that NHANES data following 2016 were not included in the analyses because weighting variables changed in the year cohorts after 2016.

The modification in weighting variables after 2016 may have unintentionally given the false impression of a temporal shift that did not truly occur. Consequently, the chosen timeframe, encompassing the years 2003 to 2016, was judiciously selected to standardize the analytical framework. This temporal constraint ensures uniformity in variable utilization and weighting procedures throughout the considered period.

### Study subjects

We included individuals aged 20 and older who completed the in-home interview. Our cohort included individuals who answered “Yes” to the question “Ever told you had cancer or malignancy” and who answered, “Lung cancer” or “Bladder cancer” at least once to the three questions “What kind of cancer” (MCQ230a – MCQ230b – MCQ230c). Since the survey only captured information on each participant's age at the first BC or LC event, we included only the first reported event in our analyses. Therefore, if a participant reported multiple BC or LC cancer events, we only considered the first event for our analyses.

Among those with a personal history of LC or BC, we designated as never smokers those individuals who answered “no” to the question “Smoked at least 100 cigarettes in life” and “Not at all” to the question “Do you now smoke cigarettes”. We designated as active smokers those individuals who answered “yes” to the question “Smoked at least 100 cigarettes in life” and “yes” to the question “Do you now smoke cigarettes”. Finally, we designated as former smokers those individuals who answered “yes” to the question “Smoked at least 100 cigarettes in life” and “not at all” to the question “Do you now smoke cigarettes”. This classification of never, active, and former smokers among individuals with a personal history of LC or BC is based on a conventional approach commonly employed to categorize smoking status in epidemiological research (16).

For former smokers, we derived the years since quitting smoking at the time of the survey using the following two questions: “how long since quit smoking cigarettes” and “Unit of measure (day/week/month/year)”. To account for the inconsistent grouping of long-term quitters in different survey cycles, individuals who stopped smoking more than 50 years ago were assigned 50 years since quitting smoking. The variable “years since quitting smoking” was then categorized into the following groups: ≤9, 10-19, 20-29, 30-39, 40-49, and ≥ 50. In this way, we could provide granular information regarding LC and BC events distribution across different years since quitting smoking categories.

Finally, to determine the duration between smoking cessation and LC or BC diagnosis, we utilized the variables “age at bladder cancer diagnosis”, “age at lung cancer diagnosis”, “age at interview”, and “years since quitting smoking”. First, we plotted the distribution of LC and BC events according to the years that elapsed between smoking cessation and LC or BC diagnosis. Therefore, we categorized the years elapsed into 10-year intervals: ≤9, 10-19, 20-29, 30-39, 40-49, and ≥ 50 years.

To estimate the number of pack-years smoked, the years of smoking cigarettes exposure were derived using the variables “age started smoking cigarettes regularly” and “age last smoked cigarettes regularly”. The “number of cigarettes smoked during the entire life” was approximated using the “number of cigarettes smoked per day when quit” and the years of smoking cigarettes exposure. The “number of cigarettes smoked during the entire life” was then converted into pack-years smoked by dividing it by 20 (the mean number of cigarettes per pack). Finally, the number of pack-years was categorized into 0.1-20 (“light smokers”), 20.1-40 (“moderate smokers”), and 40.1 or above packs-year (“heavy smokers”) (17).

## Statistical Analysis

Since data was used from the in-home interview, the sample “interview weight variable” (wtint2yr) was used to weigh the data. A proper weighting procedure was followed to construct the correct weight variable for combined NHANES Survey Cycles as indicated on the NHANES website (11). The following variables were used to define strata (sdmvstra), and cluster (sdmvpsu). We reported descriptive statistics using frequencies and percentages for categorical variables, and median with interquartile ranges (IQR) for continuous variables, accounting for the complex survey (weights, strata, and cluster). All statistical analyses were performed using STATA (Stata/SE 17.0 for Mac [Apple Silicon] Revision 13 Oct 2022, Copyright 1985-2021 StataCorp LLC), and two-sided P values < 0.05 were considered statistically significant.

## RESULTS

### Cohort and baseline characteristics

We identified 39,221 individuals older than 19 who completed NHANES surveys between 2003 and 2016, corresponding to a Weighted National Estimate (WNE) of 219,596,787 individuals. Among those, 953,386 (WNE) individuals reported a history of LC and BC. Overall, 53.6% (WNE of 510,975 individuals) of our sample reported a personal history of LC, whereas 46.4% (WNE of 442,411 individuals) reported a history of BC. For patients with LC, the median age at diagnosis was 62 (IQR 55 – 69), and the median age at the time of the survey was 67 (IQR 63 – 74). For patients with BC, the median age at diagnosis was 55 (IQR 55 – 75), and the median age at the time of the survey was 76 (IQR 66 – 80). Further demographic details are shown in Table-1.

**Table 1:** Patient baseline characteristics according to lung or bladder cancer diagnosis.

		Lung cancer	Bladder cancer	Total	p-value
		**N=99** N*=510,975	**N=98** N*=442,411	**N=197** N*=953,386	
**Gender**					**0.061**
	Male	57 (57.6%) *260,476 (51.0%)	69 (70.4%) *286,677 (64.8%)	126 (64.0%) *547,153 (57.4%)	
	Female	42 (42.4%) *250,499 (49.0%)	29 (29.6%) *155,734 (35.2%)	71 (36.0%) *406,233 (42.6%)	
**Age at the time of the survey**					**0.055**
	20-39	2 (2.0%) *14,116 (2.8%)	2 (2.0%) *7,698 (1.7%)	4 (2.0%) *21,814 (2.3%)	
	40-59	18 (18.2%) *109,641 (21.5%)	6 (6.1%) *43,572 (9.8%)	24 (12.2%) *153,213 (16.1%)	
	60-79	53 (53.5%) *295,784 (57.9%)	54 (55.1%) *262,771 (59.4%)	107 (54.3%) *558,556 (58.6%)	
	≥ 80	26 (26.3%) *91,433 (17.9%)	36 (36.7%) *128,370 (29.0%)	62 (31.5%) *219,803 (23.1%)	
**Smoking status at the time of the survey**					**0.002**
	Never Smokers	10 (10.1%) *45,265 (8.9%)	28 (28.6%) *118,436 (26.8%)	38 (19.3%) *163,701 (17.2%)	
	ACTIVE Smokers	17 (17.2%) *96,640 (18.9%)	19 (19.4%) *99,065 (22.4%)	36 (18.3%) *195,705 (20.5%)	
	Former Smokers	72 (72.7%) *369,070 (72.2%)	51 (52.0%) *224,910 (50.8%)	123 (62.4%) *593,980 (62.3%)	
**Age at lung cancer diagnosis**					
	20-39	2 (2.0%) *14,116 (2.8%)	/	2 (2.0%) *14,116 (2.8%)	
	40-59	26 (26.5%) *159,408 (32.1%)	/	26 (26.5%) *159,408 (32.1%)	
	60-79	60 (61.2%) *294,613 (59.3%)	/	60 (61.2%) *294,613 (59.3%)	
	≥ 80	10 (10.2%) *28,927 (5.8%)	/	10 (10.2%) *28,927 (5.8%)	
**Age at bladder cancer diagnosis**					
	20-39	/	1 (1.0%) *4,118 (0.9%)	1 (1.0%) *4,118 (0.9%)	
	40-59	/	29 (30.2%) *155,148 (35.5%)	29 (30.2%) *155,148 (35.5%)	
	60-79	/	47 (49.0%) *215,624 (49.3%)	47 (49.0%) *215,624 (49.3%)	
	≥ 80	/	19 (19.8%) *62,711 (14.3%)	19 (19.8%) *62,711 (14.3%)	
**Age when quit smoking**					**<0.001**
	< 20	1 (1.4%) *4,526 (1.2%)	1 (2.0%) *7,728 (3.5%)	2 (1.7%) *12,254 (2.1%)	
	20-39	6 (8.5%) *35,449 (9.7%)	11 (22.0%) *54,481 (24.8%)	17 (14.0%) *89,931 (15.3%)	
	40-59	26 (36.6%) *141,439 (38.5%)	29 (58.0%) *130,600 (59.4%)	55 (45.5%) *272,039 (46.3%)	
	60-79	38 (53.5%) *185,694 (50.6%)	9 (18.0%) *27,216 (12.3%)	47 (38.8%) *212,910 (36.3%)	
**Years since quitting smoking at the time of the survey**					**<0.001**
	0-9	34 (47.2%) *169,278 (45.9%)	7 (14.0%) *18,679 (8.5%)	41 (33.6%) *187,956 (31.9%)	
	10-19	22 (30.6%) *134,889 (36.5%)	5 (10.0%) *22,068 (10.0%)	27 (22.1%) *156,957 (26.6%)	
	20-29	7 (9.7%) *36,317 (9.8%)	12 (24.0%) *61,632 (28.0%)	19 (15.6%) *97,950 (16.6%)	
	30-39	5 (6.9%) *16,398 (4.4%)	14 (28.0%) *51,366 (23.3%)	19 (15.6%) *67,763 (11.5%)	
	40-49	4 (5.6%) *12,188 (3.3%)	7 (14.0%) *42,743 (19.4%)	11 (9.0%) *54,931 (9.3%)	
	≥ 50	0 (0.0%) *0 (0.0%)	5 (10.0%) *23,537 (10.7%)	5 (4.1%) *23,537 (4.0%)	
**Number of pack-years smoked**					**0.050**
	0/20 pack-years	13 (20.0%) *69,221 (21.2%)	19 (40.4%) *94,944 (44.9%)	32 (28.6%) *164,215 (30.5%)	
	20.1/40 pack-years	13 (20.0%) *72,689 (22.2%)	9 (19.1%) *34,766 (16.4%)	22 (19.6%) *107,455 (19.9%)	
	> 40 pack-years	39 (60.0%) *185,027 (56.6%)	19 (40.4%) *82,022(38.7%)	58 (51.8%) *267,049 (49.6%)	

Data are presented as n (%).

* = weighted national estimates

### Smoking status and Pack Year History

Among survey respondents with a history of LC, 8.9% were never smokers, 18.9% were active smokers, and 72.2% were former smokers at the time of the survey. Among survey respondents with a history of BC, 26.8% were never smokers, 22.4% were active smokers, and 50.8% were former smokers at the time of the survey. Table-2a illustrates the smoking status at the time of cancer diagnosis and at the time of the survey for LC and BC survivors. Among our cohort of LC or BC patients who were former smokers 30.5% of them had a light smoking history (< 20 pack-years), 19.9% had a medium smoking history (20-40 pack-years), and 49.6% of them had a heavy smoking history (> 40 pack-years).

**Table 2a:** Frequencies of smoking status attitude at the time of cancer diagnosis and at the time of the survey for Lung cancer survivors and Bladder cancer survivors. Data are captured in the National Health and Nutrition Examination Survey from 2003 to 2016 and they are presented as weighted national estimates (%).

Smoking Status	Lung Cancer Survivors (%)		Bladder Cancer Survivors (%)	
	**Diagnosis**	**Survey**	**Diagnosis**	**Survey**
Never Smokers	8.9	8.9	26.8	26.8
Active Smokers	24.8	72.2	2.9	50.8
Former Smokers	24.8	18.9	2.9	22.4

### Years From Quitting to Diagnosis Among Former Smokers

**Figure 1:**
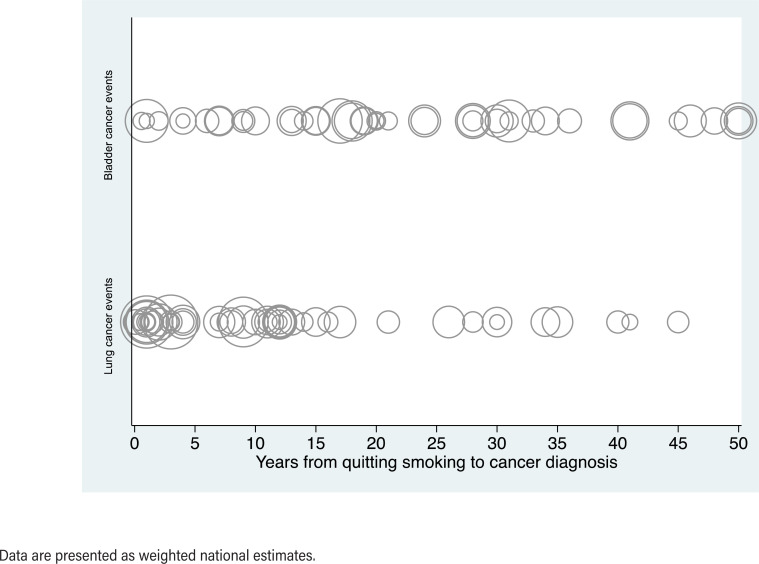
Distribution of lung or bladder cancer events affecting the population of former smokers after quitting smoking according to the years elapsed between quitting smoking and (A) Lung cancer diagnosis, and (B) Bladder cancer diagnosis, captured in the National Health and Nutrition Examination Survey from 2003 to 2016. The plot depicts the relationship between the time elapsed since smoking cessation, presented as a continuous variable, and lung or bladder cancer diagnosis. Circle-hollow markers are used to represent individual cases, with the size of the markers that is proportional to the number of lung or bladder cancer events reported at each time point.

A total of 88.3% of former smokers with a history of LC were diagnosed 0-19 years after quitting, with the majority (66.0%) being diagnosed 0-9 years after quitting. The median interval from quitting smoking to LC diagnosis was 8 years (IQR 2-12). In contrast, 49.3 % of former smokers with a history of BC were diagnosed 0-19 years after quitting. The median interval from quitting smoking to BC diagnosis was 21 years (IQR 14-33). Table-2b and Figure-1 illustrate the distribution of LC or BC events affecting the population of former smokers after quitting smoking, looking at the years elapsed from quitting smoking to LC or BC diagnosis as a categorical and continuous variable, respectively.

**Table 2b:** Distribution of Lung or Bladder cancer events affecting the population of former smokers after quitting smoking, stratified according to the latency period to cancer diagnosis. The time elapsed between smoking cessation and Lung or Bladder cancer diagnosis is stratified into 10-year categories. Data are captured in the National Health and Nutrition Examination Survey from 2003 to 2016 and they are presented as weighted national estimates (%).

Years Since Quitting Smoking	Lung Cancer Events (%)	Bladder Cancer Events (%)
0-9 years	67.0	20.0
10-19 years	21.2	29.3
20-29 years	3.8	14.5
30-39 years	5.7	15.4
40-49 years	2.3	13.1
50 years	0	7.7

## DISCUSSION

This study describes the self-reported smoking status and interval from quitting smoking to diagnosis among a sample of US adults with LC or BC. Whereas most former smokers with LC reported a short duration between quitting smoking and diagnosis (median of 8 years [IQR 2-12]), former smokers with a history of BC reported a much longer (>2.5x) interval between quitting and diagnosis (median interval of 21 years [IQR 14-33]).

While this study did not prospectively follow former smokers (it is subject to survivor bias and cannot prospectively estimate the risk at each year after quitting smoking), the findings illustrate the typical interval between quitting and diagnosis among a sample of survey respondents with LC and BC. Among respondents with BC, a comparatively large portion are diagnosed several decades after quitting smoking with an average interval of over 20 years from quitting to diagnosis. This contrasts with LC: among survey respondents with LC, most had quit less than 10 years before diagnosis. This is consistent with existing research showing a large LC risk reduction 15 years after quitting and supports consensus recommendations that LC screening can be curtailed post-quitting (18–20).

In contrast, the findings in BC support current American Urological Association guidelines that incorporate smoking history and pack-years into risk stratification for possible urothelial cancer in patients with microhematuria, with no reduction in risk categorization based on years since quitting smoking (21–23).

Biological evidence supports a prolonged latency between smoking cessation and BC diagnosis. Bladder cancer can be caused by various factors, including lifetime exposure to toxins like aromatic amines, pioglitazone medication, aristolochic acid in dietary supplements, and arsenic in drinking water, all of which can damage DNA. Cell growth, invasion, and metastasis require the acquisition of specific properties, including uncontrolled growth and cellular mobility mediated by EGF and EGFRs, modulation of cell adhesion molecules, and overproduction of angiogenic factors. As with colorectal cancer, BC pathogenesis involves a buildup of stereotyped phenotypic and molecular alterations leading to progression from adenoma to carcinoma, which can result in a prolonged latency between the genetic origins of the disease and the onset of clinically detectable symptoms. As a consequence, the disease primarily affects older adults due to the cumulative impact of long-term exposure to carcinogens and procarcinogens as well as deficient DNA repair mechanisms in this population, uncertain immune mechanisms, and local factors such as urinary retention that collectively contribute to increased disease risk (24, 25).

Furthermore, the number of former smokers is constantly increasing. As previously pointed out, it exceeded that of active smokers in the early 2000s (7). Looking at BC statistics in the US from 2005, a reduction from 19.9 (in 2005) to 16.8 (in 2019) of BC observed events rate per 100,000 persons is reported (15, 20). Seisen et al. already underlined that assuming quitting smoking leads to an immediate and progressive reduction in BC risk, the large variations in tobacco smoking prevalence only partially explain the incidence trends for BC in the US population over the past half-century. Therefore, they addressed occupational and environmental exposures and genetic predispositions as the possible explanatory of this persistence of high BC incidence (26). Our finding that large portions of BC patients are diagnosed several decades after quitting smoking suggests that the relationship between smoking and BC risk may be more complex and long-lasting.

Our study has several limitations. Firstly, recall bias may have affected the accuracy of self-reported data on medical conditions and smoking habits. Secondly, we only focused on smoking habits as a risk factor for BC and LC, excluding other known risk factors such as occupational and environmental exposure, genetic background, and infective pathogens. Additionally, our findings are based on the self-reported data of former smokers with a history of LC or BC, and only those who survived long enough to fill out the survey post-diagnosis were included. If data on those who died were available, the time from quitting smoking until cancer diagnosis could be shorter in both groups. This may affect the interpretation of our results. Furthermore, BC is typically diagnosed at an older median age than LC and has a higher survival rate. This may lead to bias in the sample, over-representing those with BC and a longer latency period. Lastly, due to differences in data collection across NHANES cycles, some approximations were made in reporting certain variables in the analysis.

Despite limitations, our study highlights an extended latency period post-smoking cessation and BC diagnosis, particularly in contrast to the analogous interval observed for lung cancer in former smokers. Our study supports ongoing BC risk even many years after quitting smoking.

## CONCLUSIONS

These results highlight the typical smoking history of individuals with lung and bladder cancer responding to a large national survey of US adults. Although there are potential sources for bias with any retrospective survey-based research, large portions of survey respondents with bladder cancer were diagnosed several decades after quitting smoking, with an average of nearly 25 years between quitting and diagnosis. This finding supports current guidelines and risk stratification models, which incorporate smoking history as a bladder cancer risk factors even many years after quitting.
